# Importance of DNA Sequencing for Abnormal Hemoglobins Detected by HPLC Screening

**DOI:** 10.4274/tjh.galenos.2020.2019.0470

**Published:** 2020-05-06

**Authors:** Duran Canatan, Abdullah Çim, Serpil Delibaş, Emel Altunsoy, Serdar Ceylaner

**Affiliations:** 1 Antalya Genetic Diseases Diagnosis Center, Antalya, Turkey; 2 Mediterranean Blood Diseases Foundation - Hemoglobinopathy Diagnosis Center, Antalya, Turkey; 3 Intergen Genetic Diseases Diagnosis Center, Ankara, Turkey

**Keywords:** Hemoglobinopathy, HPLC, DNA, Sequencing

## To the Editor,

Hemoglobinopathies are the most common health problem in Turkey. A hemoglobinopathy prevention program has been implemented by the Ministry of Health in Turkey in 33 provinces since 2003 and it spread to all 81 provinces in 2018 [1]. Our hemoglobinopathy diagnostic center has been licensed for 16 years [2]. The aim of this study was to compare the molecular genetic analysis and high-performance liquid chromatography (HPLC) results for abnormal hemoglobins. Blood samples were directed from local primary health care centers, hospitals, and laboratories in the context of premarital screening processes. Complete blood count (CBC) and HPLC methods were applied for all blood samples. Abnormal hemoglobins or abnormal bands were detected in 219 (0.67%) of 32,513 blood samples between 2013 and 2019. DNA sequencing was performed for 190 of 219 samples. Of those 190 samples, 38 were abnormal bands, 76 were HbS, 49 were HbD, 6 were HbC, and 21 were HbE.  While ten different mutations were detected in 24 cases (63.2%), they were not found for 14 (36.8%) of 38 abnormal bands (Table 1). In addition, molecular analysis confirmed 69 cases of HbS (90.8%) from among 76 HbS, 42 HbD Punjab (85.7%) in 49 HbD, 4 HbC (66.7%) in 6 HbC, and 4 HbE (19%)  in 21 HbE samples detected by HPLC.

Al-Madhani et al. [3] screened 3740 newborns and compared the results of CBC and HPLC with the molecular genetic analysis results for 290 newborns. They confirmed 26 cases of homozygous sickle cell anemia and 5 of homozygous β-thalassemia major by DNA sequencing among 31 newborns [3]. Warghade et al. [4] screened 65,779 cases for hemoglobinopathy using cation-exchange (CE)-HPLC and abnormal hemoglobin fractions were observed in 12,131 (18.44%) cases. They confirmed eight rare hemoglobin variants by beta-globin gene analysis for those samples that could not be distinguished by CE-HPLC [4]. Chen et al. screened couples of reproductive age using HPLC and reported 1.14% hemoglobinopathy in the Chinese city of Guangzhou. They reported 8 different abnormal hemoglobins by molecular techniques [5].

In the present study, the concordance of sequencing analysis with the HPLC results was 90.8% for HbS, 85.7% for HbD, 66.7% for HbC, and 19% for HbE. Interestingly, 10 different abnormal hemoglobin variants have been detected using DNA sequencing in 24 of 38 (63.2%) samples with abnormal bands. Therefore, the type of abnormal hemoglobin can be determined more precisely using molecular analysis.

In conclusion, whatever screening method is used in hemoglobinopathy diagnosis centers, all reports should include the following expression: “This is a screening test; molecular analysis should be carried out for a definite result”. Taking into account the different results obtained in screening and molecular analysis, physicians working in these centers should be offered access to molecular analysis for all abnormal hemoglobins and abnormal bands.

## Figures and Tables

**Table 1:**
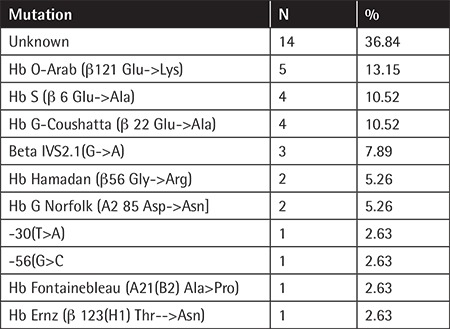
Molecular analysis of the cases with abnormal bands (n=38).
